# *Enteromorpha prolifera* Extract Improves Memory in Scopolamine-Treated Mice via Downregulating Amyloid-β Expression and Upregulating BDNF/TrkB Pathway

**DOI:** 10.3390/antiox9070620

**Published:** 2020-07-15

**Authors:** Seung Yeon Baek, Fu Yi Li, Da Hee Kim, Su Jin Kim, Mee Ree Kim

**Affiliations:** Department of Food and Nutrition, Chungnam National University, Daejeon 34134, Korea; qor7683@o.cnu.ac.kr (S.Y.B.); kaihuadouer@naver.com (F.Y.L.); ekgml6995@naver.com (D.H.K.); sulsan1003@naver.com (S.J.K.)

**Keywords:** *Enteromorpha prolifera*, antioxidant defense system, cholinergic enzymes, BDNF/TrkB pathway, scopolamine

## Abstract

*Enteromorpha prolifera*, a green alga, has long been used in food diets as well as traditional remedies in East Asia. Our preliminary study demonstrated that an ethyl acetate extract of *Enteromorpha prolifera* (EAEP) exhibited the strongest antioxidant activity compared to ethanol or water extracts. Nonetheless, there has been no report on the effect of EAEP on memory impairment due to oxidative damage. This study investigated whether EAEP could attenuate memory deficits in an oxidative stress-induced mouse model. EAEP was orally administered (50 or 100 mg/kg body weight (b.w.)) to mice and then scopolamine was administered. The oral administration of EAEP at 100 mg/kg b.w. significantly restored memory impairments induced by scopolamine, as evaluated by the Morris water maze test, and the passive avoidance test. Further, EAEP upregulated the protein expression of BDNF, p-CREB, p-TrkB, and p-Akt. Moreover, EAEP downregulated the expression of amyloid-β, tau, and APP. The regulation of cholinergic marker enzyme activities and the protection of neuronal cells from oxidative stress-induced cell death in the brain of mice via the downregulation of amyloid-β and the upregulation of the BDNF/TrkB pathway by EAEP suggest its potential as a pharmaceutical candidate to prevent neurodegenerative diseases.

## 1. Introduction

As life expectancy increases in humans, the incidence of neurodegenerative disorders in elderly people has increased. Alzheimer’s disease (AD), one of the most common types of neurodegenerative disorders, is caused by extensive oxidative stress [1,2]. Elevated oxidative stress is associated with mitochondrial dysfunction, which produces free radicals, and lipid peroxidation leading to the aggregation of β-amyloid [3]. Then, an abnormally excessive accumulation of β-amyloid forms oligomers and plaques that cause a progressive decline in cognition and degeneration of the cholinergic nervous system pathology [4,5].

Disruption of the cholinergic nervous system in the hippocampus and brain cortex reduces the levels of acetylcholine (ACh) and choline acetyltransferase (ChAT), and conversely, increases the levels of acetylcholinesterase (AChE), which catalyzes choline esters, leading to memory impairment and behavioral disorders [4,5,6]. Scopolamine, a blocker of muscarinic ACh receptors, is used to induce oxidative stress, reduce hippocampal volume, and dysregulate the cholinergic neuronal pathway in rodents and humans [7,8]. In addition, scopolamine reduced the expression of the brain-derived neurotrophic factor (BDNF)/tyrosine kinase (TrkB) signaling pathway, which plays a crucial role in memory processes [9,10,11,12]. BDNF, a neurotrophic factor, activates TrkB that is a BDNF receptor and enhances synaptic plasticity, memory formation, and the persistence of memory storage [11,12,13]. Hence, approaches to the treatment and prevention of Alzheimer’s disease (AD) have been studied by investigating candidates that possess not only antioxidative effects but also regulate the cholinergic system and the BDNF/TrkB pathway by administering plant-derived extracts to animal models of AD [10,14,15].

Many species of marine algae have been used as food and traditional medicines in Eastern countries and, more recently, in Europe and America [16]. A lower incidence of neurodegenerative diseases has been reported in East Asia, where people consume high amounts of fish and marine algae compared to those in Europe [17,18]. Marine algae contain abundant nutrients and diverse phytochemicals, such as polysaccharides, phlorotannins, protein hydrolysates, and photosynthetic pigments [19]. Marine-derived natural compounds exert various biochemical effects, including antioxidant [20,21], anti-viral [22], anti-inflammatory [23,24], anti-diabetic [25], and anti-allergic properties [26]. Therefore, there has been growing interest in marine algae as functional foods and nutraceuticals with potential beneficial health effects as sources of antioxidants to reduce the risk of neurodegenerative diseases.

*Enteromorpha prolifera* (EP), a green alga, is grown and cultivated in seashores worldwide, particularly in Korea, Japan, and China [27,28,29]. An ancient document reported that EP was used not only as food but also as a natural medicine for treating epistaxis and inflammation, fever, and hydrops fetalis [29,30]. EP contains abundant nutrients, such as dietary fiber, vitamins, minerals, and polyunsaturated fatty acids [31]. Furthermore, the phytochemicals in EP were reported to contain chlorophyll, phycocyanin, carotenoids, flavonoids, and phenolic compounds [31,32,33]. Our preliminary study demonstrated that an ethyl acetate extract of *Enteromorpha prolifera* (EAEP) exhibited the strongest antioxidant activity compared to ethanol or water extracts [34]. Nonetheless, there has been no report of the protective action of EP extracts against neurodegenerative diseases.

This study investigated whether an extract of *Enteromorpha prolifera* improved oxidative stress-induced memory dysfunction by regulating cholinergic enzymes and activating the antioxidant enzyme system and the BDNF/TrkB pathway.

## 2. Materials and Methods

### 2.1. Materials

Scopolamine hydrobromide, tacrine (9-amino-1,2,3,4-tetrahydroacridine hydrochloride hydrate), l-glutamic acid (glutamate), dimethyl sulfoxide (DMSO), sodium dodecyl sulfate (SDS), nicotinamide adenine dinucleotide 2′-phosphate reduced tetrasodium salt hydrate (NADPH), 5,5′-dithibis-2-nitrobenzoic acid (DTNB), glutathione reductase (GR, type 3 from baker’s yeast), l-glutathione (GSH), naringin, thiobarbituric acid (TBA), diphenyl-2-picrylhydrazyl (DPPH), oxidized glutathione (GSSG), cytochrome C, and cumene-OOH were purchased from Sigma-Aldrich Chemical Co. (St. Louis, MO, USA). Ethylenediaminetetraacetic acid (EDTA) was purchased from Junsesei Chemical Co., Ltd. (Tokyo, Japan). BDNF was obtained from Santa Cruz Biotechnology, Inc. (Paso Robles, CA, USA). Antibodies against p-TrkB, p-Akt, p-tau, and β-amyloid were purchased from Cell Signaling Technology, Inc. (Beverly, MA, USA). All other chemicals were purchased from Sigma-Aldrich Chemical Co. All reagents were of analytical grade.

### 2.2. Preparation of Ethyl Acetate Extract of Enteromorpha prolifera (EAEP)

EAEP (Songwonfood, Seosan, Korea) was prepared according to a modified method of a previously reported process [34,35]. Briefly, lyophilized *Enteromorpha prolifera* (200 g) was extracted with 95% ethanol in a bath sonicator for one day, and then the mixture was filtered using Whatman filter paper (No. 2). The process was repeated three times. The whole filtrate was concentrated using a rotary evaporator (Rikakikai Co., Tokyo, Japan). The concentrate was added to ethyl acetate and distilled water (1:1, *v/v*), and the ethyl acetate layer was separated and evaporated. The dried residue of ethyl acetate extract (2.6 g) was dissolved in dimethyl sulfoxide (DMSO) and soybean oil.

### 2.3. Animals and Experimental Induction-Scopolamine

**Figure 1:**
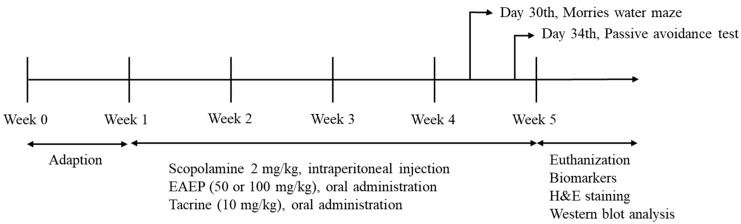
The schedule of the experiment.

Six-week-old male ICR mice were obtained from Raonbio Co. (Daejeon, Korea). The mice were adjusted at constant temperature and humidity (25.0 ± 2.0 °C, 55 ± 10%), with a 12:12 h light/dark cycle. The experimental groups and schedule diagram are shown in Table 1 and Figure 1, respectively. The mice were given free access to feed and water and weighed once a week. After one week of adjustment, the mice were randomly divided into six groups of nine mice each and treated as shown in Table 1. Briefly, 2 mg/kg body weight scopolamine dissolved in saline was injected intraperitoneally daily for four weeks. EAEP was dissolved in corn oil with 1% DMSO. Tacrine (9-amino-1,2,3,4-tetrahydroacridine hydrochloride hydrate) was dissolved in 0.9% NaCl. EAEP and tacrine were administered orally daily 30 min after the scopolamine injection for four weeks. In the fourth week, EAEP or tacrine was administered one hour before conducting the water maze test (WM) and the passive avoidance test (PA). Then, scopolamine was injected 30 min before the WM and PA. All mice care protocols and experiments were approved by the Animal Experimental Care Center of Chungnam National University (Daejeon, Korea). The animal experiments were carried out in compliance with the guidelines in the National Institutes of Health Guide for the Care and Use of Laboratory Animals (Registration No. 2019A-CNU-093).

### 2.4. Tissue Preparation and Collection

Whole mouse brains were homogenized with nine volumes of homogenization buffer (20 mM phosphate buffer containing 0.1 M KCl, 1 mM EDTA, and 0.5% Triton X-100) then centrifuged at 14,000 rpm at 4 °C for 10 min. The supernatant was used for biochemical assays and the homogenate was used for the TBA assay. The supernatant was stored as −70 °C until use.

### 2.5. Morris Water Maze Test

The Morris water maze test was conducted to evaluate cognitive changes from EAEP treatment. A black circular pool (150 cm in diameter and 60 cm deep) was used, as in a previous study [36]. The pool was filled with water at 23 ± 1 °C and divided into quadrants. The Morris water maze test was conducted on six days before the end of the experiment.

On the first experimental day, the mice were trained for 120 s in the absence of the transparent platform (10 cm diameter) for adaptation to the pool. Over four consecutive days, the mice were dropped onto one of the quadrants, with the platform placed 1 cm under the surface of the water. The starting point was changed in a different order each day. The time needed to find the platform was recorded on each of the four days. When a mouse reached the platform, it was permitted to remain on it for 10 s. If a mouse did not find the platform within 120 s, it was placed on the platform for 10 s. The mice were dried with a towel after each trial. On the last day of the trial, the platform was hidden 5 cm under the surface of the water. The first time the mice passed the platform and the number of times they crossed the platform were recorded as the escape latency and cross times, respectively, using a video camera (TGCAM-2000STA, Sambo Electronic Co., Ltd., Seoul, Korea).

### 2.6. Passive Avoidance Test

Passive avoidance was evaluated in a light and dark chamber (Jung Bio & Plant Co., Ltd., Seoul, Korea) as previously reported [37,38]. EAEP and tacrine were injected orally, then scopolamine was injected one hour later, and the passive avoidance test was conducted 30 min later. The passive avoidance test was conducted on two days just before the end of the experiment. For data acquisition, the mice were initially placed in the light chamber with a closed door for 1 min, then the door was opened. The time of moving to the dark chamber was recorded. When the mice entered the dark chamber, the door was closed. Then, the mice were given a 0.5 mA electronic shock through stainless steel rods for 5 s. After 24 h, the mice were placed into the light chamber again, and the time of entering the dark chamber was recorded as the latency time.

### 2.7. Protein Determination

Protein was measured with a Bio-Rad protein assay dye reagent [39]. Supernatant from the mice brains was mixed with 1:5 diluted dye reagent and incubated at room temperature for 10 min. The absorbance of the mixture was measured by a spectrophotometer at 595 nm. Bovine serum albumin (BSA) was used to generate a standard curve in the range of 0.2–1.5 mg/mL.

### 2.8. Measurement of Acetylcholinesterase (AChE) and Choline Acetyltransferase (ChAT) Activity

AChE activity was measured by a colorimetric reaction with an acetylthiocholine iodide substrate as reported in a previous study [40]. Brain supernatant was mixed with 0.1 M phosphate buffer, 10 mM Ellman’s reagent, and 75 mM acetylthiochloride iodide. The absorbance of the mixture was measured by a spectrophotometer at 410 nm for 5 min, at 1 min intervals. ChAT activity was analyzed using a kit (Elabscience Biotechnology Co., Ltd., Houston, TX, USA), following the manufacturer’s instructions.

### 2.9. Measurement of Lipid Peroxide Concentration in Brain

The brains were placed on ice and homogenized with 50 mM sodium phosphate buffer using a tissue homogenizer with a Teflon pestle (Dupont, Wilmington, DE, USA). Homogenate (1 mL) was mixed with 1 mL of 8.1% SDS, 2 mL of 20% acetic acid, and 1 mL of 0.75% TBA and boiled for 30 min. The absorbance of the malondialdehyde (MDA)-TBA adduct formed in the supernatant was measured at 532 nm as previously described. The MDA value was compared to a standard curve that was prepared with tetramethoxypropane (TMP) and expressed as the TBA value.

### 2.10. Measurement of Antioxidant Enzyme Activities in Brain

The glutathione (GSH), glutathione reductase (GR), and glutathione peroxidase (GPx) concentrations in the brain homogenates were measured using the following method. The GSH activity was determined by mixing with 0.1 M potassium phosphate buffer, 10 mM DTNB, and 5 mM NADPH, equilibrated for 1 min by adding 1 unit of glutathione reductase, and the absorbance was measured at 412 nm using a spectrophotometer. GSH (0.04 mM) was used to generate a standard curve. The antioxidant enzyme activity was calculated in 1 g of protein determined by the Bradford method using BSA as a standard. The GR activity was analyzed by reacting with 26.98 mM EDTA in 0.1 M Tris-HCl buffer, 66.0 mM GSSG, and 9.18 mM NADPH, and the absorbance was measured with a spectrophotometer at 340 nm. The GPx activity was measured by reacting with 0.1 M sodium phosphate buffer, 5 mM NADPH, 100 mM GSH, and 1 unit of glutathione reductase. After 3 min, 100 mM cumene-OOH was added to the mixture. The OD was measured by a spectrophotometer at 340 nm.

### 2.11. Histological Examination

For histological staining, the mice were perfusion-fixed, and the brains were isolated. The brains were stored in 4% paraformaldehyde and then embedded in paraffin. Then, 4 μm sections were cut using a microtome (Leica RM 2165, Leica Microsystems GmbH, Wetzlar, Germany). The paraffin sections were deparaffinized, rehydrated, and stained with hematoxylin and eosin (H&E). The histopathological changes were assessed under a light microscope.

### 2.12. Western Blot Analysis

The mice were euthanized after treatment, and their brains were removed. The brain tissues from both hemispheres were promptly excised and homogenized in ice-cold Radioimmunoprecipitation assay (RIPA) buffer containing protease inhibitor cocktails. The protein concentration in the brain tissue was determined as above. The sample was subjected to 7.5–15% SDS-polyacrylamide gel separation under reducing conditions, transferred to a polyvinylidene difluoride transfer membrane (Bio-Rad Laboratories Inc., Hercules, CA, USA) for 30–45 min. The membrane was blocked with 5% BSA in Tris-buffered saline containing 0.1% Tween-20 (TBST) for 2–3 h at room temperature. Then, the membrane was incubated overnight for two days at 4 °C with BDNF, p-TrkB, p-Akt (ser 473), p-tau, β-amyloid, and β–actin primary antibodies. After five washes with TBST, the blots were incubated with a horseradish peroxidase-conjugated secondary antibody (Cell Signaling Technology) in 5% BSA containing TBST (1:4000 dilution) for 2–3 h at room temperature. Subsequently, the membrane was developed using an enhanced chemiluminescence detection kit (Advansta, San Jose, CA, USA) and exposed to X-ray film (AGFA, Antwerp, Belgium). The relative intensity of each protein was quantified by using Image J software (NIH Image, Bethesda, MD, USA).

### 2.13. Statistical Analysis

All the results are expressed as the mean ± S.E.M. All statistical analyses were performed using the SPSS 24.0 program (SPSS Inc., Chicago, IL, USA) and GraphPad Prism 8 (GraphPad Software Inc., San Diego, CA, USA) software. The data were analyzed by one-way analysis of variance (ANOVA), followed by the LSD (least-significant difference) test, and a post-hoc comparison was made using Duncan’s multiple range test to further analyze the differences between the groups. Statistical significance was indicated as * *p* < 0.05, ** *p* < 0.01, or *** *p* < 0.001 compared to the control, and as # *p* < 0.05, ## *p* < 0.01, or ### *p* < 0.001 compared to the scopolamine-induced group.

## 3. Results

### 3.1. Weight of the Body and Brains of Mice Treated with EAEP

The effect of EAEP on body weight and brain weight in mice is shown in Table 2. The body weight was measured once a week during the experimental period. The brain weight was measured after euthanization and collection. The body weight of the mice was increased at the end of the experiment. However, there was no significant difference in body weight or brain weight between the scopolamine-induced groups and the controls. These results are very important because they mean that the administration of EAEP has no cytotoxicity in scopolamine-treated mice.

### 3.2. Effect of EAEP on Spatial Learning Ability in the Morris Water Maze

**Figure 2:**
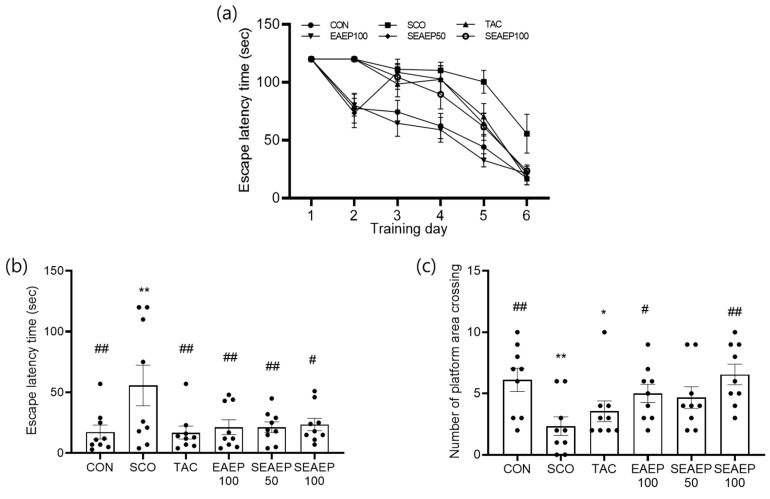
Effect of ethyl acetate extract of *Enteromorpha prolifera* (EAEP) on memory-enhancing in scopolamine-induced mice. The escape latency time during the training days (**a**), escape latency time (**b**), and the number of platform area crossings (**c**) in Morris water maze test were measured in the mice. CON: Non-treated group; SCO: Scopolamine 2 mg/kg-treated group; TAC: Scopolamine 2 mg/kg + tacrine 10 mg/kg-treated group; EAEP 100: EAEP 100 mg/kg-treated group; SEAEP 50: Scopolamine 2 mg/kg + EAEP 50 mg/kg-treated group; SEAEP 100: Scopolamine 2 mg/kg + EP 100 mg/kg-treated group. All data are mean ± S.E.M. (*n* = 9/group). Statistical significance was indicated as * *p* < 0.05, or ** *p* < 0.01 compared to the control, and as # *p* < 0.05, or ## *p* < 0.01 compared to the scopolamine-induced group.

To investigate the effect of EAEP on spatial learning ability in scopolamine-induced mice, the Morris water maze test was carried out sequentially for six days. For this purpose, the effect of EAEP (50 or 100 mg/kg) on the escape latency time (ELT) and the times of platform area crossing (TPAC) in scopolamine-induced memory-impaired mice was examined. As shown in Figure 2a,b, the ELT of the scopolamine-induced mice was significantly longer than that of the control mice during all trial sessions (*p* < 0.01), indicating the successful induction of dementia in the mouse model. The oral administration of EAEP (50 or 100 mg/kg) significantly restored the ELT elongated by scopolamine, similar to the results in mice treated with tacrine, an inhibitor of scopolamine. On the final day of the training trial sessions, the normal control mice took about 17.3 s to find the platform, whereas the scopolamine-treated mice took about 55.7 s. The administration of EAEP reversed the extended ELT. The ELT of EAEP at 50 or 100 mg/kg was 21.3 and 23.6 s, respectively. TPAC was examined by removing the platform to evaluate spatial learning memory. As shown in Figure 2c, the TPAC of the scopolamine-treated mice was significantly decreased compared to that of the normal controls. However, the administration of EAEP (50 or 100 mg/kg) dose-dependently increased the TPAC. The TPAC of mice treated with EAEP (100 mg/kg) was even higher than that of the tacrine-treated group (*p* < 0.01), similar to that of the normal controls. These results showed that EAEP influenced spatial memory and learning in scopolamine-induced mice. Thus, EAEP may exert an attenuating effect on scopolamine-induced memory impairment in mice.

### 3.3. Effect of EAEP on Memory Ability in the Passive Avoidance Test

**Figure 3:**
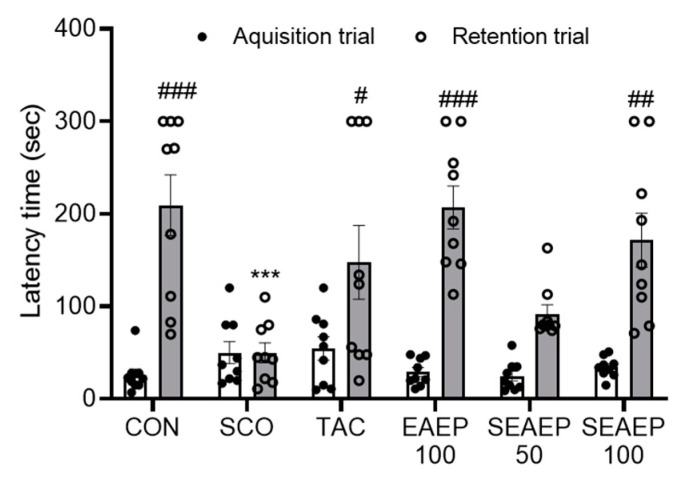
Effect of ethyl acetate extract of *Enteromorpha prolifera* (EAEP) on memory-enhancing in scopolamine-induced mice. Latency time in the passive avoidance test was measured in mice. CON: Non-treated group; SCO: Scopolamine 2 mg/kg-treated group; TAC: Scopolamine 2 mg/kg + tacrine 10 mg/kg-treated group; EAEP 100: EAEP 100 mg/kg-treated group; SEAEP 50: Scopolamine 2 mg/kg + EAEP 50 mg/kg-treated group; SEAEP 100: Scopolamine 2 mg/kg + EAEP 100 mg/kg-treated group. All data are mean ± S.E.M. (*n* = 9/group). Statistical significance was indicated as *** *p* < 0.001 compared to the control, and as # *p* < 0.05, ## *p* < 0.01, or ### *p* < 0.001 compared to the scopolamine-induced group.

Next, the passive avoidance test was conducted over two days just before the end of the experiment. To evaluate the effect of EAEP on memory and learning ability, the acquisition trial and retention trial times were measured (Figure 3). The acquisition trial time was similar among all the experimental groups. The latency time in the scopolamine-treated mice decreased significantly compared to the normal control mice. The administration of EAEP (50 or 100 mg/kg) exhibited a dose-dependent mitigating effect. Furthermore, the ameliorating effect of EAEP (100 mg/kg) on memory impairment was better than that of tacrine. Separately, EAEP alone had no effect on the behavior of normal mice. These results provide evidence that EAEP may have a protective effect and improve the behavioral dysfunction of scopolamine-induced dementia.

### 3.4. Effect of EAEP on Acetylcholinesterase (AChE) and Choline Acetyltransferase (ChAT) Activity

**Figure 4:**
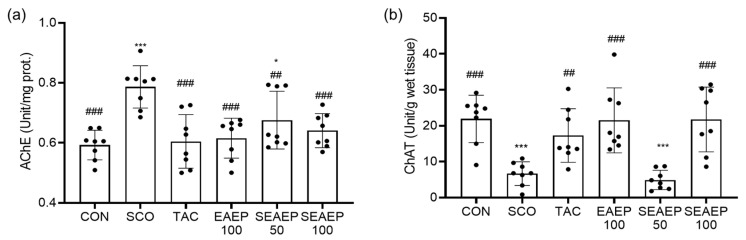
Effect of ethyl acetate extract of *Enteromorpha prolifera* (EAEP) on the cholinergic nervous system in scopolamine-induced mice. AChE (acethylcholine esterase) activity (**a**) and ChAT (choline acetyl transferase) activity (**b**) were measured in mice. CON: Non-treated group; SCO: Scopolamine 2 mg/kg-treated group; TAC: Scopolamine 2 mg/kg + tacrine 10 mg/kg-treated group; EAEP 100: EAEP 100 mg/kg-treated group; SEAEP 50: Scopolamine 2 mg/kg + EAEP 50 mg/kg-treated group; SEAEP 100: Scopolamine 2 mg/kg + EAEP 100 mg/kg-treated group. All data are mean ± S.E.M. (*n* = 8/group). Statistical significance was indicated as * *p* < 0.05, or *** *p* < 0.001 compared to the control, and as ## *p* < 0.01, or ### *p* < 0.001 compared to the scopolamine-induced group.

Acetylcholine (Ach), an important neurotransmitter in learning and memory, is hydrolyzed by AChE, whereas it is synthesized by ChAT in the brain. To verify that the administration of EAEP regulated AChE or ChAT in the brain of mice, the AChE and ChAT activities were measured. As shown in Figure 4a, the activity of AChE in scopolamine-treated mice was the highest among all groups (*p* < 0.05), suggesting that a dysfunction of the cholinergic nervous system may facilitate the progression of dementia. In contrast, the administration of EAEP significantly returned AChE activity to a normal level, in a dose-dependent manner (*p* < 0.01). Separately, the administration of EAEP (100 mg/mL)-only had no significant effect on brain AChE activity.

As shown in Figure 4b, the activity of ChAT in scopolamine-treated mice decreased significantly compared to that of normal control mice (*p* < 0.001). The activity of ChAT in EAEP-treated mice (100 mg/kg) recovered to the normal level, similar to that in tacrine-treated mice. Tacrine, an AChE inhibitor, was one of the first medicinal materials for alleviating and treating Alzheimer’s disease to be consumed widely [41]. Thus, EAEP treatment prevented the scopolamine-induced effects on AChE activity and reduction in ChAT activity, indicating that the neuroprotective effect of EAEP on scopolamine-induced cholinergic dysregulation may be mediated through the cholinergic nervous system.

### 3.5. Effect of EAEP Antioxidant Enzyme Activities and Lipid Peroxide Contents

**Figure 5:**
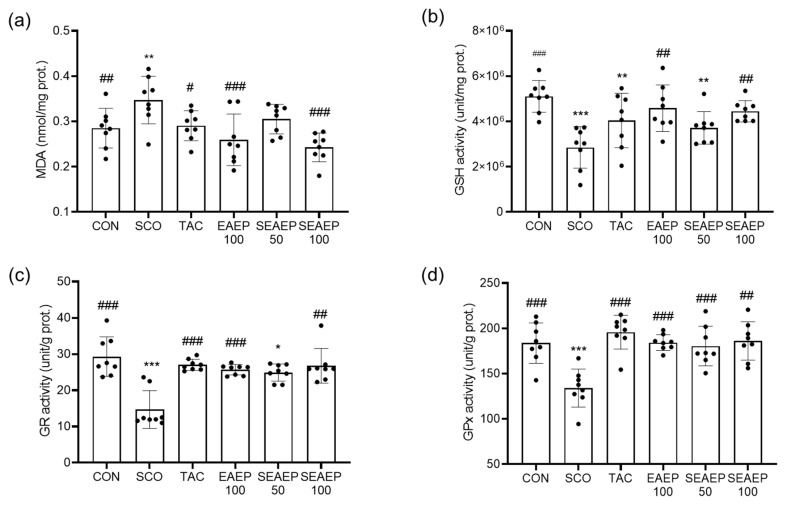
Effect of ethyl acetate extract of *Enteromorpha prolifera* (EAEP) on antioxidant enzymes in scopolamine-induced mice. The MDA (malondialdehyde) level (**a**), GSH (glutathione) activity (**b**), GR (glutathione reductase) activity (**c**), and GPx (glutathione peroxidase) activity (**d**) were measured in mice. CON: Non-treated group; SCO: Scopolamine 2 mg/kg-treated group; TAC: Scopolamine 2 mg/kg + tacrine 10 mg/kg-treated group; EAEP 100: EAEP 100 mg/kg-treated group; SEAEP 50: Scopolamine 2 mg/kg + EAEP 50 mg/kg-treated group; SEAEP 100: Scopolamine 2 mg/kg + EAEP 100 mg/kg-treated group. All data are mean ± S.E.M. (*n* = 8/group). Statistical significance was indicated as * *p* < 0.05, ** *p* < 0.01, or *** *p* < 0.001 compared to the control, and as # *p* < 0.05, ## *p* < 0.01, or ### *p* < 0.001 compared to the scopolamine-induced group.

Malondialdehyde, known as the final product of lipid peroxidation, plays a causative role in oxidative injury in tissues when its levels increase [42]. To further elucidate the underlying mechanism of the protective effect of EAEP in scopolamine-induced dementia, the levels of malondialdehyde (MDA) and antioxidant enzymes GSH, GPx, and GR were assessed in the mice brains. As shown in Figure 5a, the oxidative stress of scopolamine injection caused lipid peroxidation in the brains of the mice, where the MDA levels in the scopolamine-treated mice were significantly higher than those in normal control mice (*p* < 0.01). The administration of EAEP prevented lipid peroxidation from oxidative stress in the brain of scopolamine-induced mice. The MDA levels in mice treated with EAEP (50 or 100 mg/kg) mice were decreased in a dose-dependent manner (*p* < 0.05). Furthermore, EAEP (100 mg/kg) was more effective than tacrine treatment in preventing the incremental increases in MDA (*p* < 0.05).

Scopolamine alone significantly decreased the total GSH levels (Figure 5b). The administration of EAEP (50 or 100 mg/kg) restored the total GSH levels (*p* < 0.05). The activities of GPx and GR were the lowest in the scopolamine-treated mice (*p* < 0.001). The protective effect of EAEP (50 or 100 mg/kg) was similar to that of tacrine (Figure 5c,d). Therefore, these results suggest that EAEP may protect the brain from oxidative injury by scopolamine, by preventing scopolamine-induced oxidative stress.

### 3.6. Effect of EAEP on Hippocampal Neurons in the CA1 and CA3 Regions

**Figure 6:**
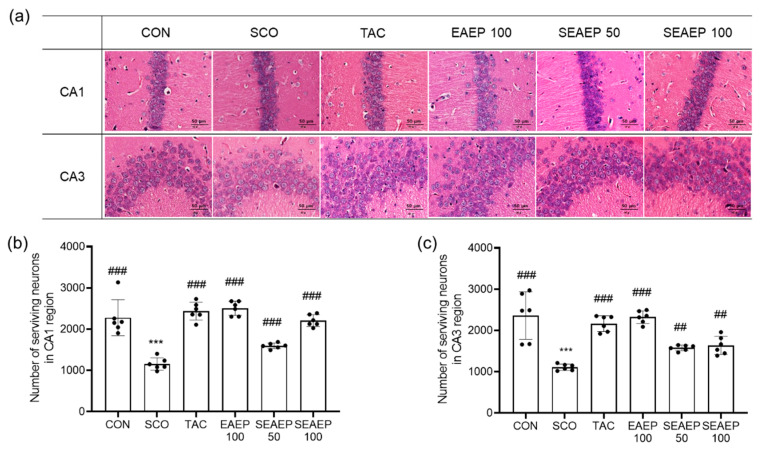
Effect of ethyl acetate extract of *Enteromorpha prolifera* (EAEP) on hippocampal neurons in CA1 and CA3 region. Histological sections with hematoxylin and eosin (H&E) staining of the CA1 and CA3 regions in the hippocampi of mice (**a**), the number of surviving neurons in the CA1 region (**b**), and the number of surviving neurons in the CA3 region (**c**). CON: Non-treated group; SCO: Scopolamine 2 mg/kg-treated group; TAC: Scopolamine 2 mg/kg + tacrine 10 mg/kg-treated group; EAEP 100: EAEP 100 mg/kg treated-group; SEAEP 50: Scopolamine 2 mg/kg + EAEP 50 mg/kg-treated group; SEAEP 100: Scopolamine 2 mg/kg + EAEP 100 mg/kg-treated group. All data are mean ± S.E.M. (*n* = 6/group). Statistical significance was indicated as *** *p* < 0.001 compared to the control, and as ## *p* < 0.01, or ### *p* < 0.001 compared to the scopolamine-induced group.

Dementia induced by scopolamine is known to cause extensive injury to pyramidal cells in the hippocampal CA1 and CA3 regions. Scopolamine-induced cell damage leads to neuronal degeneration, exhibited as a pericellular halo and spongiform changes of neutrophils [43]. The effect of EAEP on morphological changes and neuronal degeneration in the hippocampus of scopolamine-treated mice is shown in Figure 6. The number of degenerated neurons was significantly increased in the CA1 and CA3 regions of scopolamine-treated mice (*p* < 0.001). The administration of EAEP (50 or 100 mg/kg) improved the neuronal degeneration in scopolamine-induced oxidative injury in a dose-dependent manner (*p* < 0.001). In the CA1 region of scopolamine-induced mice treated with EAEP (100 mg/kg), the amount of neuronal degradation was restored to that in normal control mice. These results suggest that EAEP attenuated neuronal injury from oxidative stress induced by scopolamine.

### 3.7. Effect of EAEP on β-Amyloid and Tau Expression

**Figure 7:**
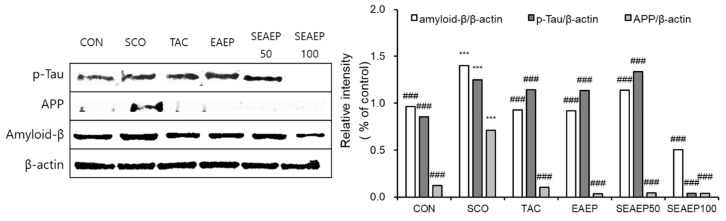
Effect of ethyl acetate extract of *Enteromorpha prolifera* (EAEP) on expression of p-Tau, APP, and Amyloid β in brain tissue from mice treated with scopolamine. The supernatant of brain homogenate was subjected to SDS-PAGE, and a western blot analysis was performed using each specific antibody against p-Tau, APP, Amyloid β, or β-actin. β-Actin was used as loading controls. The results are representative of three experiments conducted under each condition. CON: Non-treated group; SCO: Scopolamine 2 mg/kg-treated group; TAC: Scopolamine 2 mg/kg + tacrine 10 mg/kg-treated group; EAEP: EAEP 100 mg/kg-treated group; SEAEP 50: Scopolamine 2 mg/kg + EAEP 50 mg/kg-treated group; SEAEP 100: Scopolamine 2 mg/kg + EAEP 100 mg/kg-treated group. All data are mean ± S.E.M. (*n* = 3/group). Statistical significance was indicated as *** *p* < 0.001 compared to the control, and as ### *p* < 0.001 compared to the scopolamine-induced group.

In Alzheimer’s disease, the expression of β-amyloid (Aβ) and tau in the brain cause synaptic dysfunction, neuron loss, and neurodegeneration, causing cognitive deficits and memory impairment [2,44]. First, the expression of Aβ and phosphorylated-tau in scopolamine-treated mice was significantly increased, as shown in Figure 7 (*p* < 0.001), suggesting scopolamine-induced dementia. Meanwhile, the administration of EAEP (50 or 100 mg/kg) significantly prevented the expression of Aβ and tau in scopolamine-treated mice. The expression of Aβ and tau in mice treated with EAEP (100 mg/kg) was enhanced significantly compared to that in mice treated with tacrine (*p* < 0.01). These results indicate that EAEP could attenuate protein expression related to scopolamine-induced dementia.

### 3.8. Effect of EAEP on Protein Expression via BDNF/TrkB/Akt Pathway

**Figure 8:**
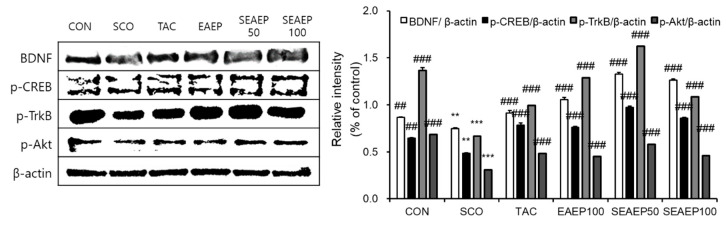
Effect of ethyl acetate extract of *Enteromorpha prolifera* (EAEP) on expression of BDNF, p-CREB, p-TrkB, and p-Akt in brain tissue from mice treated with scopolamine. The supernatant of brain homogenate was subjected to SDS-PAGE, and a western blot analysis was performed using each specific antibody against BDNF, p-CREB, p-TrkB, and p-Akt or β-actin. β-Actin was used as loading controls. The results are representative of three experiments conducted under each condition. CON: Non-treated group; SCO: Scopolamine 2 mg/kg-treated group; TAC: Scopolamine 2 mg/kg + tacrine 10 mg/kg-treated group; EAEP 100: EAEP 100 mg/kg-treated group; SEAEP 50: Scopolamine 2 mg/kg + EAEP 50 mg/kg-treated group; SEAEP 100: Scopolamine 2 mg/kg + EAEP 100 mg/kg-treated group. All data are mean ± S.E.M. (*n* = 3/group). Statistical significance was indicated as ** *p* < 0.01, or *** *p* < 0.001 compared to the control, and as ## *p* < 0.01, or ### *p* < 0.001 compared to the scopolamine-induced group.

BDNF, a neurotrophin, enhances synaptic plasticity, memory formation, and the persistence of memory storage [13]. TrkB, a BDNF receptor, leads to the dimerization and autophosphorylation of tyrosine residues and subsequently activates cytoplasmic signaling when bound to BDNF [9]. Akt, a downstream factor in PI3K, plays a crucial role in enhancing the translation of BDNF [45]. In this regard, the effect of EAEP on the expression of the BDNF/TrkB/Akt pathway was evaluated in the brain (Figure 8). The level of BDNF and p-CREB in the hippocampus of scopolamine-treated mice was decreased and the administration of EAEP (50 or 100 mg/kg) increased the expression (*p* < 0.01). Likewise, phosphorylated-TrkB (p-TrkB) was reduced by scopolamine treatment, but EAEP significantly restored the level of p-TrkB. Furthermore, the expression of p-Akt in scopolamine-treated mice was significantly decreased (*p* < 0.001) and the administration of EAEP to scopolamine-treated mice increased the expression p-Akt (*p* < 0.001). Thus, these results demonstrated that the effect of EAEP may be partially related to the regulation of learning and memory-related proteins involved in the BDNF/TrkB/Akt-signaling pathway in the brain.

## 4. Discussion

Marine algae are abundant and various species have been recently investigated as potential cosmeceutical and nutraceutical agents, and for therapeutic applications [46]. Algae are recognized as a source of important bioactive ingredients, such as antioxidants, proteins, vitamins, minerals, soluble dietary fibers, polyunsaturated fatty acids, polysaccharides, and photosynthetic pigments [19,31,47,48,49,50]. Moreover, epidemiological studies showed that there was an association between algae consumption and a lower incidence of neurodegenerative diseases [16,19,46,47].

*Enteromorpha prolifera* (EP), a green alga, grows in seashores, particularly in East Asia. From ancient times, EP has been used in food diets as well as in traditional remedies for treating epistaxis and inflammation, fever, and hydrops fetalis [27,28,29,30]. EP contains various nutrients, such as vitamins, phytochemicals, and polyunsaturated fatty acids and abundant phytochemicals, including carotenoids, flavonoids, phenolic compounds, phycocyanin, and chlorophyll, which were reported to have biological activities including antioxidant, anti-inflammatory, and anti-diabetes effects [31,33,35]. However, studies on the neuroprotective effect of EP extract have been not reported.

The accumulation of reactive oxygen species (ROS) and reactive nitrogen species (RNS), and the interaction between these reactive species causes lipid peroxidation, protein oxidation, DNA damage, and, ultimately, neuronal cell death [51]. In many studies, oxidative stress, the imbalance between pro-oxidant and antioxidant homeostasis leading to the generation of toxic ROS, has been implicated in the pathogenesis of most neurodegenerative disorders [1,3,11,52,53]. Specifically, oxidative stress has been found as indicator preceding the cardinal neuropathology and was chronologically associated with other key features of AD, such as metabolic, mitochondrial, and cell-cycle abnormalities [53]. These disorders cause the clinical symptoms of AD, including memory deficit, cognitive dysfunction, delusions, apathy, depression, and behavior disorders [3,53,54].

In the present study, the scopolamine-induced dementia model was used to explore the neuroprotective effect of EP extracted with ethyl acetate (EAEP). The oral administration of EAEP (50 or 100 mg/kg) attenuated scopolamine-induced memory impairment in the mouse model. First, the neuroprotective effect of EAEP on memory and learning was assessed by the Morris water maze test, which evaluated spatial learning ability [36]. EAEP significantly improved scopolamine-induced memory deficits by reducing the lengthened escape latency time and increasing the number of crossings over the platform site, indicating that the administration of EAEP markedly enhanced spatial learning ability [55]. This suggests that EAEP ameliorated scopolamine-induced dementia. In addition, the enhancing effect of EAEP on memory was further examined by a passive avoidance test, which investigated the effect of treatments on three phases of the memorization process: Learning acquisition, consolidation, and retrieval [37]. Notably, EAEP increased the retention time that was decreased by scopolamine treatment. These results suggest that the memory-enhancing effect of EAEP in scopolamine-induced memory impairment may be associated with modulation of the cholinergic nervous system. The most noticeable biological change in AD patients was the reduction in ACh levels in the hippocampus and cortex of the brain [11,12,53,54]. Thus, the mechanism of the memory-enhancing effect of EAEP on the cholinergic nervous system was assessed in brain tissue. Cholinergic dysfunction occurs in cholinergic signaling disorders in attention, spatial memory, and cognitive processes related to the mechanism of ACh, which plays an important role in encoding and retrieval in memory processing [56]. In the scopolamine-treated model, the cholinergic nervous system is disabled, increasing the level of AChE, an enzyme that breaks down Ach, and decreasing the level of ChAT, an ACh-synthesizing enzyme [8]. Since the termination of cholinergic transmission is influenced by the level of ACh, AChE-inhibiting drugs, such as tacrine, donepezil hydrochloride, and rivastigmine have been used to produce positive results in patients with dementia [57]. For this reason, tacrine, which is an inhibitor of AChE, has been used to improve cognitive and memory deficits by increasing brain cholinergic activity [43]. In our study, the administration of EAEP reversed the increase in AChE activity as well as the decline in ChAT activity in scopolamine-treated brains. Therefore, the cholinergic-regulating effect of EAEP on scopolamine-induced cholinergic dysfunction may be associated with behavioral changes.

In addition, scopolamine has been reported to induce oxidative stress in brain tissue, further increase lipid peroxidation, and reduce the activities of antioxidant enzymes [4,58,59]. Consistent with this hypothesis, the present study showed that the level of lipid peroxidation in scopolamine-treated mice increased, whereas the activities of antioxidant enzymes, including GSH, GPx, and GR, decreased. The administration of EAEP significantly improved the antioxidant defense system by suppressing lipid peroxidation and activating antioxidant enzymes against scopolamine-induced oxidative stress. Increases in oxidative stress were reported to show interaction between the activities of ChAT and GPx [43], consistent with our results [4,14,43]. Various antioxidant compounds with neuroprotective effects, including flavonoids, phlorotannins, sulfated polysaccharides, carotenoids, and sterols, have been detected in different marine algae [19,23,32,33,48,52]. Antioxidants from EAEP, including dihydroactinidiolide, canthaxanthin, and fucoxanthin, which are derived from carotenoids, and dimethylsulphoniopropionate (DMSP), which is derived from methionine, were detected by LC/MS (liquid-chromatography mass spectrometry) analysis [34]. EAEP significantly prevented against the scopolamine-induced oxidative stress of neurons in the hippocampal CA1 and CA3 regions. These results provide evidence that the neuroprotective effect of EAEP on scopolamine-induced oxidative stress may be primarily associated with its antioxidant action. Besides, the levels of β-amyloid (Aβ) and tau, which are indicator proteins of AD, were overexpressed, suggesting that scopolamine induced memory deficits. Meanwhile, EAEP (100 mg/kg) significantly suppressed the scopolamine-induced expression of Aβ and tau. The accumulation of Aβ has neurotoxic effects by inducing the generation of ROS, leading to neuronal dysfunction and eventually cell death, as shown in Figure 6 [51,52,53]. EAEP not only increased the levels of ACh in the brain but also reduced Aβ deposits, protecting neurons from neurodegeneration [44].

Of note, the effect of EAEP on the BDNF/TrkB/Akt pathway was examined, since the BDNF/TrkB/Akt pathway is known to modulate the growth and complexity of dendrites and play a critical role in learning and memory formation processes [9,11,12,59,60]. BDNF, a neurotrophic factor, activates TrkB, a BDNF receptor, and enhances synaptic plasticity, memory formation, and the persistence of memory storage. The expression of BDNF increases protein synthesis by enhancing translation initiation via multiple signaling pathways, such as Akt [61]. In previous studies, the BDNF/TrkB/Akt-signaling pathway was activated by memory behavioral training for spatial reference and working memory [12,45]. Here, scopolamine decreased the expression levels of BDNF, p-TrkB, and p-Akt in the brain, whereas EAEP significantly restored the scopolamine-induced memory deficits. The complexity of various antioxidants in EAEP contributed to its efficacy against memory deficit, although this study did not confirm the active compound(s). Further study is required to identify the active compounds in the marine algae extract that are involved in improving scopolamine-mediated memory impairment. Moreover, further studies of other animal models are necessary to confirm the effect of memory improvements in EAEP for potential preventive and clinical purposes [62,63].

In summary, we found that EAEP could prevent scopolamine-induced memory impairment by regulating the cholinergic nervous system, the antioxidant defense system, and the BDNF/TrkB-signaling pathway. Taken together, EAEP can be a potential source of abundant phytochemicals, improving memory impairment, and it may be a candidate for functional health food and pharmaceuticals for neuroprotection.

## 5. Conclusions

We demonstrated that EAEP protected against scopolamine-induced memory impairment, and that the neuroprotective effects of EAEP may regulate the cholinergic nervous system, the antioxidant defense system, and the BDNF/TrkB/Akt-signaling pathway. Our study suggests that EAEP may be a candidate for neuroprotective treatments.

## Figures and Tables

**Table 1:** Experimental design.

Group	Treatment		Head
	Oral Administration	Intraperitoneal Injection	
CON	Corn oil	0.9% NaCl	9
SCO	Corn oil	2 mg/kg scopolamine	9
TAC	10 mg/kg tacrine	2 mg/kg scopolamine	9
EAEP 100	100 mg/kg EAEP	0.9% NaCl	9
SEAEP 50	50 mg/kg EAEP	2 mg/kg scopolamine	9
SEAEP 100	100 mg/kg EAEP	2 mg/kg scopolamine	9

CON: Non-treated group; SCO: Scopolamine 2 mg/kg-treated group; TAC: Scopolamine 2 mg/kg + tacrine 10 mg/kg-treated group; EAEP: EAEP 100 mg/kg-treated group; SEAEP 50: Scopolamine 2 mg/kg + EAEP 50 mg/kg-treated group; SEAEP 100: Scopolamine 2 mg/kg + EAEP 100 mg/kg-treated group.

**Table 2:** Body and brain weights of the mice.

Group		CON	SCO	TAC	EAEP 100	SEAEP 50	SEAEP 100
Weight of body (g)	0 week	22.81 ± 0.17	23.23 ± 0.18	23.66 ± 0.18	23.15 ± 0.28	23.31 ± 0.18	23.05 ± 0.16
	1 week	35.22 ± 0.68	36.33 ± 0.41	36.25 ± 0.59	35.93 ± 0.43	36.73 ± 0.55	34.81 ± 0.72
	2 weeks	35.32 ± 0.75	36.13 ± 0.46	35.91 ± 0.81	36.19 ± 0.43	36.55 ± 0.39	34.36 ± 0.62
	3 weeks	36.62 ± 0.56	36.22 ± 0.50	36.42 ± 0.95	36.59 ± 0.48	37.27 ± 0.52	34.96 ± 0.69
	4 weeks	38.22 ± 0.67	37.65 ± 0.47	37.93 ± 1.05	37.52 ± 0.58	38.83 ± 0.53	37.48 ± 0.75
	5 weeks	38.22 ± 0.67	37.41 ± 0.47	38.51 ± 1.16	37.50 ± 0.54	38.68 ± 0.63	37.08 ± 0.95
Weight of brain (g)		0.44 ± 0.02	0.43 ± 0.02	0.45 ± 0.01	0.48 ± 0.02	0.48 ± 0.01	0.47 ± 0.02

CON: Non-treated group; SCO: Scopolamine 2 mg/kg-treated group; TAC: Scopolamine 2 mg/kg + tacrine 10 mg/kg-treated group; EAEP 100: EAEP 100 mg/kg-treated group; SEAEP 50: Scopolamine 2 mg/kg + EAEP 50 mg/kg-treated group; SEAEP 100: Scopolamine 2 mg/kg + EAEP 100 mg/kg-treated group. All data are mean ± S.E.M. (*n* = 9/group). There was no significant difference between the groups.
